# *In vitro* Methods for the Development and Analysis of Human Primary Airway Epithelia

**DOI:** 10.3389/fphar.2018.01176

**Published:** 2018-10-26

**Authors:** Ambra Gianotti, Livia Delpiano, Emanuela Caci

**Affiliations:** U.O.C. Genetica Medica, IRCSS Istituto Giannina Gaslini, Genoa, Italy

**Keywords:** cystic fibrosis, bronchus, airway epithelial cells culture, short-circuit current recording, transepithelial electrical resistance measurement, periciliary mucus properties

## Abstract

Cystic fibrosis (CF) is a chronic disease caused by mutations in the CF transmembrane conductance regulator (*CFTR*) gene, which encodes for a channel expressed at the apical surface of epithelial tissues. Defective chloride and bicarbonate secretion, arising from CFTR mutations, cause a multi-organ disease. In the airways, impaired ion transport results in a thick mucus, dehydration of the periciliar region and bacterial infections. Over the last years, basic research has sustained a great effort to identify therapies that are able to correct defective CFTR. For this purpose, *in vitro* cell models have played a key role in the study of mechanisms of the disease and to assess CFTR modulator therapies. Cultures of human primary bronchial epithelia are considered a physiologically relevant disease model due to their ability to maintain most of the morphological and functional characteristics of the airway epithelium *in vivo*. Despite their value, these cells are limited by the availability of human lung tissue and by the complexity of the culture procedure. However, primary human nasal cells can be considered as an alternative model for the study of CF pathophysiology since they are easier to obtain and recapitulate the properties of bronchial cultures. Over the years, several groups have optimized a protocol with key steps to culture and fully amplify differentiated primary airway epithelia. Our approach provides epithelia monolayers grown on porous filters, characterized by high transepithelial electrical resistance and an electrical potential difference. These parameters are required to perform electrophysiological experiments devoted to the study of ion transport mechanisms in airway epithelia. The aim of this study was to describe different methods to expand and differentiate isolated cells into fully polarized monolayers of airway epithelium, in order to provide an optimized protocol to support physiopathology analysis and to evaluate therapeutic strategies.

## Introduction

Cystic fibrosis (CF) is an autosomal recessive disease caused by mutations in the gene coding for the protein cystic fibrosis transmembrane conductance regulator (CFTR). CFTR is a chloride channel expressed at the apical surface of secretory epithelia where it plays an important role in salt and fluid homeostasis (Saint-Criq and Gray, 2017).

The consequences of mutated CFTR are most important in the airways. The resulting defective chloride secretion, coupled to enhanced sodium absorption, impairs normal mucociliary clearance due to dehydration of the airway surface and consequently causes uncontrolled inflammation and chronic bacterial infections (Castellani and Assael, 2017).

Recently, bicarbonate has emerged as another important anion that contributes to the genesis of CF lung disease due to its role in mucus release (Quinton, 2008; Borowitz, 2015).

Over the last years, basic research has sustained a great effort to define a strategy to identify novel therapies that correct the basic defects responsible for CFTR loss-of-function.

Studies have demonstrated that pharmacological rescue of the CFTR protein, in the presence of small molecules called correctors and potentiators, can repair folding and gating defects (Pedemonte et al., 2005; Okiyoneda et al., 2013; Van Goor et al., 2009, 2011).

The most successful compounds, ivacaftor and lumacaftor developed by Vertex Pharmaceuticals company, have been approved for treatment of CF patients (Wainwright et al., 2015).

Drug discovery is supported by *in vitro* cell models that offer the opportunity to study epithelium physiology, the alterations caused by mutated CFTR, and the efficacy of therapies that aim to correct basic CF defects.

*In vitro* cell models for airway epithelium studies have been mostly based on primary human bronchial epithelial cells (hereafter termed HBEC), obtained from CF lung resection.

HBEC provide the ideal tool since they exhibit several of the morphological and functional defects of airway epithelia. Despite their value, it is difficult to acquire a large amount of cells and they can only be grown for 4–5 passages before reverting to a poorly differentiated phenotype.

Primary nasal epithelial cells (hereafter termed HNEC) have been recently proposed as an alternative method to HBEC culture.

HNEC are easy to collect by nasal brushing and recapitulate the properties of HBEC cultures (McDougall et al., 2008; Mosler et al., 2008). Furthermore, this model is very useful to predict the clinical treatment efficacy in patients (Pranke et al., 2017).

Several research groups have optimized protocols that allow isolation, expansion, and differentiation of primary HBEC and HNEC (Fulcher et al., 2005; Yaghi et al., 2010; Neuberger et al., 2011; Müller et al., 2013; Stokes et al., 2014).

Primary cells have a very limited proliferative capacity *in vitro* and it is possible to culture for at least five-six passages before noting a slowing down of cell growth. To overcome this problem, culturing of bronchial and nasal epithelial cells under CRC conditions, namely irradiated feeder cells and the RhoA kinase inhibitor Y, enhances cell growth and lifespan while preserving electrophysiological and morphological properties (Gentzsch et al., 2017).

The aim of this article was to present a detailed protocol, optimized in our laboratory, for culture and differentiation of airway epithelia. This method results in large-scale production of isolated HBEC and HNEC and fully differentiated epithelia that exhibit the morphological and functional defects of CF airways. Therefore, these cell models are very useful for improving our knowledge about physiopathology mechanisms involved in CF and to support therapeutics strategies.

Our approach is based on the isolation of airway cells from bronchi or nasal brushings obtained from CF and non-CF subjects. Then, isolated cells are cultured and expanded with a high proliferation rate using a proliferative serum-free medium. This step is followed by epithelial cell differentiation on permeable supports, whose ion transport properties can be evaluated with electrophysiological techniques (Galietta et al., 1998; Scudieri et al., 2012).

For this purpose, we review two powerful methods for ion transport measurements underlining the application, advantages, and limits. These include Ussing chamber and Trans-Epithelial Electrical Resistance (TEER) techniques (Li et al., 2004; Srinivasan et al., 2015).

Moreover, we describe a cell culture protocol to achieve a fully differentiated mucociliary airway epithelium to study the properties of periciliary mucus considering its important involvement in the CF pathology. Indeed, the reduction of fluid secretion in CF alters the composition of the airway surface fluid (ASL) and induces the production of mucus with rheological properties making it inadequate for normal physiology (Gianotti et al., 2016).

Finally, our culture protocol allows morphological and functional characteristics of the airway epithelium to be reproduced *in vivo*, supporting the study of CF physiopathology and therapeutic strategies.

## Materials and Equipments

### Cell Culture

• Petri dishes, culture flasks, Pasteur pipette, physiological saline solution.
• Snapwell permeable support: 3801 (Corning, Tewksbury, MA, United States).
• HTS Transwell permeable support: 3379 (Corning).
• HBSS (hepes buffered saline solution): hepes (20 mM, Sigma #H4034, Saint Louis, MO, United States), sodium chloride (121.0 mM, Sigma #S9888), potassium chloride (2.7 mM, Sigma #P3911), glucose (9.4 mM, Sigma #G7528), sodium phosphate dibasic (7.2 mM, Sigma #S7907), phenol red (1:1000 of 0.5% stock solution, Sigma #P5530). Adjust pH to 7.4 and bring to volume; sterilize with a filter and store at +4°C.
• Protease solution: protease powder of *Streptomyces griseus* XIV (0.3 g, Sigma #P5147) dissolved in 130 ml HBSS and 30 ml Ham’s F12 w/o L-glut. Prepare before using, sterilize with a filter and store at +4°C.
• Ca^2+^/Mg^2+^ – free phosphate buffered saline (PBS).
• Ca^2+^/Mg^2+^ – Dulbecco’s phosphate buffer saline (D-PBS).
• Trypsin solution: Ca^2+^/Mg^2+^-free phosphate buffered saline (PBS) containing 0.05% trypsin and 0.02% EDTA solution.
• Rat tail collagen solution: rat tail collagen (1 mg, Sigma #C7661) dissolved in 1 ml acetic acid 0.1 M.
• SMAD inhibitors cocktail: A 83-01 (1 μM, Sigma #SML0788), DMH1 (1 μM, Sigma #D8946).
• Rock inhibitor: Y-27632 2HCl (5 μM, Sigma #SCM075).
• Proliferative serum-free medium: See Table 1 and Supplementary Materials. Sterilize with a filter and store at +4°C. This medium may be frozen. Protect from light with aluminum foil.

**Table 1:** Proliferative serum-free medium.

Compound	Final concentration
LHC basal medium (Life Technology #12677-019)	500 ml
RPMI 1640	500 ml
Stock 4	5 ml/L
Stock 11	0.5 ml/L
Calcium stock	40.6 μM
Insulin stock	0.4325 μM
Transferrin stock	61.5 μM
EGF stock	0.403 nM
P/E stock	2.5 ml/L
RA/T3 stock	0.05 ml/L
Trace element solution	5 ml/L
HC stock	0.1 μM
Bovine pituitary extract (BPE) (Gibco #13028-014)	25 mg/L
Penicillin	100 U/ml
Streptomycin	100 μg/ml
L-glutamine	2 mM
• Cryomedium: prepare a solution of fetal bovine serum (FBS) with 10% DMSO. Prepare before using, store at +4°C for no longer than 1 month.
• Medium with serum to neutralize trypsin: DMEM High glucose w/o L-glut, Ham’s F12 w/o L-glut, FBS 10%, penicillin (100 U/ml), streptomycin (100 μg/ml), L-glutamine (2 mM). Sterilize with a filter and store at +4°C.
• Differentiation serum-supplemented medium: See Table 2 and Supplementary Materials. Sterilize with a filter and store at +4°C for no longer than 3 weeks. This medium may not be frozen, freezing seems to destroy the ability to induce differentiation and the appearance of electrical resistance. Protect from light with aluminum foil.

**Table 2:** Differentiation serum-supplemented medium.

Compound	Final concentration
DMEM High glucose w/o L-glut	500 ml
Ham’s F12 w/o L-glut	500 ml
Insulin stock	0.4325 μM
Transferrin stock	61.5 μM
P/E stock	2.5 ml/L
RA/T3 stock	0.1 ml/L
HC stock	0.2 μM
Bovine pituitary extract (BPE) (Gibco #13028-014)	25 mg/L
New Zealand Serum	20 ml/L
Penicillin	100 U/ml
Streptomycin	100 μg/ml
L-glutamine	2 mM
• Differentiation serum-free medium: See Table 3 and Supplementary Materials. Sterilize with a filter and store at +4°C for no longer than 3 weeks. This medium cannot be frozen, freezing seems to destroy ability to induce differentiation and the appearance of electrical resistance. Protect from light with aluminum foil.

**Table 3:** Differentiation serum-free medium.

Compound	Final concentration
DMEM High glucose w/o L-glut	500 ml
Ham’s F12 w/o L-glut	500 ml
Insulin stock	0.87 μM
Transferrin stock	123 μM
EGF stock	0.806 nM
Epinephrine stock	2.73 μM
RA stock	0.099 μM
T3 stock	0.01 μM
HC stock	1.4 μM
Bovine pituitary extract (BPE) (Gibco #13028-014)	50 mg/L
BSA stock	22.58 μM
Penicillin	100 U/ml
Streptomycin	100 μg/ml
L-glutamine	2 mM

### Electrophysiological Equipment

• Epithelial voltohmmeter: EVOM2 [World precision Instrument (WPI), Sarasota, FL, United States].
• Voltage-clamp (DVD-1000 voltage/current clamp, WPI Instruments).
• PowerLab 4/25 (AD Instruments, Colorado Springs, CO, United States).
• Ag/AgCl electrodes: EK1 (WPI Instruments).
• Agar salt bridges (1 M potassium chloride in 2% agar).
• Ussing chambers (Vertical Diffusion Chamber, Warner Instruments, Hamden, CT, United States).
• Latex tubes for perfusion.
• Ringer’s Solution for Ussing chamber: sodium chloride (126 mM, Sigma #S9888), potassium dihydrogen phosphate (0.38 mM, Sigma #NIST200B), potassium phosphate dibasic trihydrate (2.13 mM, Sigma #P9666), magnesium sulfate heptahydrate (1 mM, Sigma #M9397), calcium chloride dihydrate (1 mM, Sigma #C8106), glucose (10 mM, Sigma #G7528), sodium bicarbonate (24 mM, Sigma #S5761), phenol red (1:1000 of 0.5% stock solution, Sigma #P5530). Sterilize with a filter and store at +4°C.
• Coon’s modified Ham’s F-12 medium for TEER: hepes (20 mM), FBS 10%, penicillin (100 U/ml), streptomycin (100 μg/ml), L-glutamine (2 mM). Sterilize with a filter and store at +4°C.
• Amiloride hydrochloride (Sigma #A7410), CPT-cAMP (Sigma #C3912), forskolin (Selleckchem #S2449, Munich, Germany), genistein (Sigma # G6649), lumacaftor (VX-809, Selleckchem #S1565), ivacaftor (VX-770, Selleckchem #S1144), CFTR-inh172 (Selleckchem #S7139), UTP (Sigma #U6625), ATP (Sigma #A9187), PPQ-102 (Sigma #219677).

## Stepwise Procedures

### Primary HBEC and HNEC Culture

In this step, we describe a cell culture protocol which can provide, from a relatively little amount of sample, a large number of polarized bronchial and nasal epithelia useful for the study of airway physiopathology and predicting *in vivo* drug efficacy.

This method is based on two distinct phases:

1. Expansion of HBEC and HNEC using a proliferative serum-free medium. This culture medium allows the expansion of airway epithelial cells with a high proliferation rate and a low density growth for at least five passages before cell proliferation slows down. Cumulative population doubling level at fifth passage is approximately 33.
2. Differentiation of epithelia on permeable supports using a “air liquid interface” (ALI) condition:
  • serum-supplemented medium condition: cells differentiate into fully polarized monolayers of airway epithelium in order to perform electrophysiological experiments.
  • serum-free medium condition: cells form a fully differentiated mucociliary airway epithelium useful for the study of periciliary fluid properties of airways (Hajj et al., 2007).

#### Isolation and Expansion of Primary HBEC Derived From CF and Non-CF Donors Following Lung Transplantation

1. Dissect and wash the mainstem human bronchus, derived from CF and non-CF individuals undergoing lung transplant, with physiological saline solution. Transfer the sample into a saline solution at +4°C in the laboratory and process within 4 h.
2. To clean the bronchus of the mucus, wash the sample twice in a 50 ml conical tube containing physiological saline solution.
3. Place the bronchus overnight at +4°C in protease XIV solution in order to separate the epithelial cell layer from the underlying connective tissue.
4. Remove the bronchus gently from the protease XIV solution and place it in a 100-mm Petri-dish containing physiological saline solution. Collect epithelial cells by vigorously flushing the bronchial lumen with physiological saline solution (Figure 1).

**FIGURE 1:**
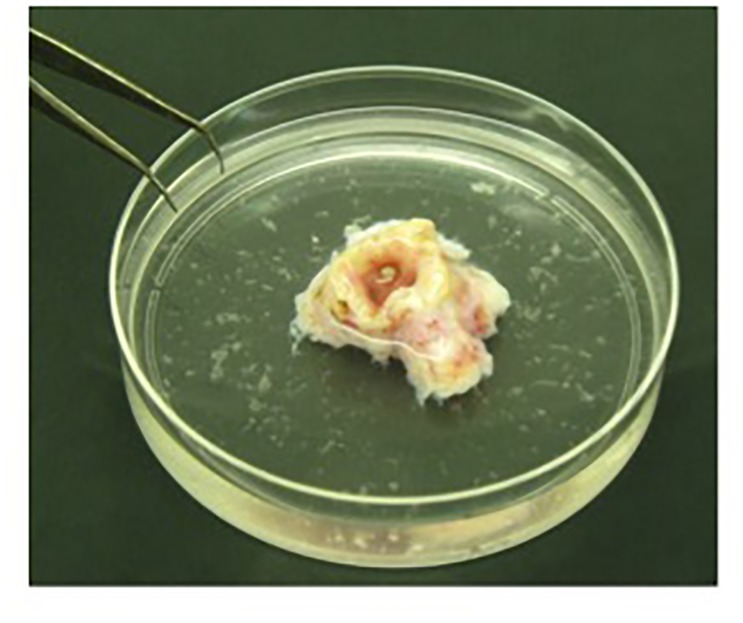
Detached epithelial cells isolated from bronchus. Bronchus is placed in a 100-mm Petri-dish and epithelial cells are collected by flushing the bronchial lumen vigorously with physiological saline solution.
5. Pellet the detached epithelial cells by centrifugation at 900 rpm for 10 min.
6. Resuspend the cells in 3 ml of trypsin solution, gently pipette five times using a 1-ml pipette tip and incubate at 37°C for 5 min.
7. Neutralize trypsin solution by adding an equal volume of medium with serum.
8. Add proliferative medium to bring the final volume to 35 ml.
9. Count the cells to determine the cell yield and then centrifuge at 900 rpm for 10 min.
10. Coat a T150 flask with 100 μl/cm^2^ of rat tail collagen solution and place in an incubator at 37°C (5% CO_2_) for at least 15 min or up to 6 h. Before use, carefully aspirate the collagen solution with a Pasteur pipette.
11. Resuspend the pellet in proliferative medium in order to seed approximately 8000 cells/cm^2^ in the collagen-coated flask and incubate at 37°C (5% CO_2_).
12. To expand the cell culture, replace the proliferative medium every 24 h until the cell density reaches 70% confluence.
13. When the bronchus was derived from a CF patient, treat the medium with antibiotics for the first 6 days to eradicate bacterial contamination. The mixture of antibiotics is designed based on the results of the antibiogram of the bacteria isolated from the patient’s most recent expectorate. If the antibiogram is not available use the following cocktail: colistimethate sodium, piperacillin/tazobactam. Use a specific antibiotic cocktail at a 10X concentration for the first 48 h, then reduce every two days by 1:2 to minimize cell toxicity (Table 4).

**Table 4:** Antibiotic and antifungal dose concentrations suggested for CF cell culture treatment.

Antibiotic	Dose 10x	Antibiotics type	Indications
Tobramycin	8 μg/mL	Aminoglycoside	Gram-negative Bacteria
Amikacin	25 μg/mL	Aminoglycoside	Gram-negative Bacteria
Gentamicin	40 μg/mL	Aminoglycoside	Gram-negative Bacteria
Piperacillin-Tazobactam	50 μg/mL	Beta-lactamase inhibitors	Gram-positive Bacteria
Ticarcillin	250 μg/mL	Beta-lactamase inhibitors	Gram-positive Bacteria
Imipenem	2000 μg/mL	Beta-lactamase inhibitors	Gram-positive Bacteria
Meropenem	250 μg/mL	Beta-lactamase inhibitors	Gram-positive Bacteria
Ceftazidime	250 μg/mL	Beta-lactamase inhibitors	Gram-positive Bacteria
Oxazolidinone (Linezolid)	2 mg/mL	Inhibitor on the ribosomal 50S	Gram-positive Bacteria
Teicoplanin	60 μg/mL	Semisynthetic glycopeptide	Gram-positive Bacteria
Vancomycin	50 μg/mL	Glycopeptide	Gram-positive Bacteria
Ciprofloxacin	25 μg/mL	Fluoroquinolone	Gram-positive and negative Bacteria
Levofloxacin	100 μg/mL	Fluoroquinolone	Gram-positive and negative Bacteria
Trimethoprim + Sulfamethoxazole	25 μg/mL	Sulfonamides	Gram-positive and negative Bacteria
Colistimethate sodium	50 U/mL	Polymyxin	Multi-drug resistant Bacteria
Amphotericin B	25 μg/mL	Macrocyclic polyene	Antifungal
  To overcome fungal contamination, treat the medium with 2.5 μg/ml of amphotericin B for at least the first 7 days of culture.
14. When the cell density reaches 70% confluence, harvest HBEC for further expansion or freeze aliquots for future use.
15. To detach HBEC, remove the proliferative media and add 15 ml of warm Ca^2+^Mg^2+^-free phosphate buffer solution (PBS) and place at 37°C (5% CO_2_) for 6 min.
16. Remove PBS and add 2.4 ml of trypsin solution and incubate at 37°C (5% CO_2_) until detachment.
17. Neutralize the trypsin solution by adding an equal volume of medium with serum.
18. Add 20 ml PBS and gently swirl the flask to collect the cells.
19. Transfer the cell suspension to a 50 ml conical tube.
20. Count the cells to determine the cell yield. The expected yield for one T150 flask is ∼6 million cells.
21. Centrifuge at 1500 rpm for 5 min.
22. Resuspend the pellet in 10 ml of proliferative medium and split the cell culture between 3 collagen-coated T150 flasks for further expansion.
23. Replace the growth media every 24 h during the cell expansion phase.
24. Freeze cells every cell splitting in order to have a bio-bank for every passage.
25. For this, resuspend cell culture in cryoprotective-medium at a density of 2 million cells/ml. Place 1 ml of this culture into each cryovial and freeze in a cell freezing container at -80°C for at least 24 h before transferring to a liquid nitrogen tank for long-term storage.
26. To thaw frozen HBEC, place the cryovial in a 37°C water bath for 2 min and then transfer the cell culture into a 15 ml conical tube containing proliferative medium. Centrifuge at 1500 rpm for 5 min.
27. Coat 2 T75 flasks with 100 μl/cm^2^ of rat tail collagen solution and place in an incubator at 37°C (5% CO_2_) for at least 15 min or up to 6 h. Before use, aspire carefully the collagen solution with a Pasteur pipette.
28. Resuspend the pellet in 8 ml proliferative medium and split the culture in 2 collagen-coated T75 flasks already containing 8 ml growth medium/flask. Replace medium every 2 days and maintain culture as described above.

#### Isolation and Expansion of Primary HNEC Derived from CF and Non-CF Donors Following Nasal Brushing

1. Collect HNEC by nasal brushing of both nostrils. The nasal brushing is performed without local anesthesia after nasal washings with physiological saline solution, in order to remove the mucus (two washes a day in the previous week and a wash immediately before nasal cells collection).
2. Use a soft sterile interdental brush with 2.5–3 mm bristles scraping along the middle portion of the inferior turbinate with anterior-posterior rotational movements in each nostril, under direct vision.
3. Carefully monitor patient for vital and minor signs, comfort and pain.
4. Place brush in a 15 ml conical tube containing 9 ml of Ca^2+^Mg^2+^-D-PBS, transfer the sample to the laboratory and process within 4 h.
5. Place the sample in a 37°C water bath for 20 min and then centrifuge at 900 rpm for 5 min to detach all cells from brush.
6. Coat 1 T25 flask with 100 μl/cm^2^ of rat tail collagen solution and place in incubator at 37°C (5% CO_2_) for at least 15 min until 6 h. Before use, carefully aspirate the collagen solution with a Pasteur pipette.
7. Carefully remove the brush and resuspend the cell pellet in 6 ml of proliferative medium and seed it in the coated T25 flask. To improve growth rate and to extend the number of population doublings *in vitro*, the culture medium is supplemented with a SMAD and Rock inhibitors cocktail required for the conditional reprogramming and immortalization of human epithelial cells (Palechor-Ceron et al., 2013; Mou et al., 2016).
8. Maintain cultures as previously described for HBEC.

#### Generation of Differentiated Nasal and Bronchial Epithelia With Serum-Supplemented Medium

1. Culture and detach HBEC and HNEC on the fourth passage when the cell density reaches 90% confluence.
2. Count the cells to determine the cell yield. The expected yield for T75 and T150 flasks is ∼4 and 8 million cells, respectively.
3. Seed 500.000 cells/insert on Snapwell permeable supports in 500 μl proliferative medium. Add 2 ml proliferative medium on the basolateral side.
4. Alternately, seed 200.000 cells/insert on HTS Transwell permeable supports in 300 μl proliferative medium. Add 25 ml proliferative medium in the bottom well.
5. After 24 h, replace the proliferative medium with differentiation serum-supplemented medium. Add 500 μl and 2 ml of medium on the apical and basolateral sides, respectively, on a Snapwell insert.
6. Alternately, add 200 μl to the apical side and 25 ml in the bottom well of the HTS Transwell insert.
7. Replace medium daily on both sides of the permeable supports until the sixth day (liquid-liquid culture).
8. Check differentiation of cells into a tight epithelium by measuring transepithelial electrical resistance and potential difference with an epithelial voltohmmeter EVOM2.
9. Good values measured in Snapwell supports are ∼-40 mV and ∼-30 mV for CF and non-CF epithelia, respectively, and 1000–1700 Ω for both genotypes.
10. Subsequently, totally remove the apical medium (ALI condition) and replace medium every two days only in the basolateral side. This condition favors a fully differentiated epithelium.
11. Maintain HBEC and HNEC culture for 2–3 weeks.

#### Generation of Differentiated Nasal and Bronchial Epithelia With Serum-Free Medium

1. Culture and detach HBEC and HNEC on the fourth passage when cell density reaches 65% confluence.
2. Coat the Snapwell support with 200 μl of rat tail collagen solution and place in an incubator at 37°C (5% CO_2_) for at least 15 min or up to 6 h. Before use, carefully aspirate the collagen solution with a Pasteur pipette.
3. Count and seed 150.000 cells/insert on Snapwell supports in 500 μl proliferative medium. Add 2 ml proliferative medium to the basolateral side.
4. After 24 h, replace the proliferative medium with fresh medium at both sides of the permeable support.
5. After 24 h, replace the proliferative medium with serum-free differentiation medium only in the basolateral side (ALI condition).
6. Replace medium every 2 days only on the basolateral side of the permeable support until the 15^th^ day.
7. Check differentiation of cells into a tight epithelium by measuring transepithelial electrical resistance and potential difference with an epithelial voltohmmeter EVOM2.
8. Good values are ∼-8 for CF and ∼-5 for non-CF epithelia, respectively, and 600–800 Ω for both genotypes.
9. Maintain the ALI culture condition epithelia for 4–5 weeks.

#### HBEC and HNEC Monolayers Application

Well differentiated HBEC and HNEC epithelia are useful to support the following studies:

1. Electrophysiological experiments aimed at the study of ion transport mechanisms in airway epithelia.
2. Analysis of protein expression with immunofluorescence or Western Blot techniques.
3. Gene expression analysis by RT-qPCR, microarray or RNAseq.
4. Investigation of innate defense mechanisms and response to proinflammatory stimuli.

#### Important Remarks

1. During the initial isolation from bronchus, columnar cells with rapidly beating cilia should be observed indicating good cell viability. Beating cilia cells will disappear in the culture condition over time. The day after plating, isolated cells from bronchus, many red blood cells, and fibroblasts should be present in the culture and disappear after washing the flask with physiological saline solution. Small spots of epithelial cells should be observed.
2. HBEC from CF patients are frequently infected with antibiotic-resistant organisms. Particular care has been given to eradicate bacterial and fungal contamination with appropriate treatments.
3. During cell expansion HBEC and HNEC can be split up to four times to expand the number of cells available for functional studies. Further expansion may frequently lead to worsening of epithelia performance.
4. During expansion, special care must be taken when splitting cells into new flasks that the cell density reaches no more than 70% confluence. A higher density could induce a squamous differentiation (Sacco et al., 1992) that debars desired morphological and functional properties (Figure 2).

**FIGURE 2:**
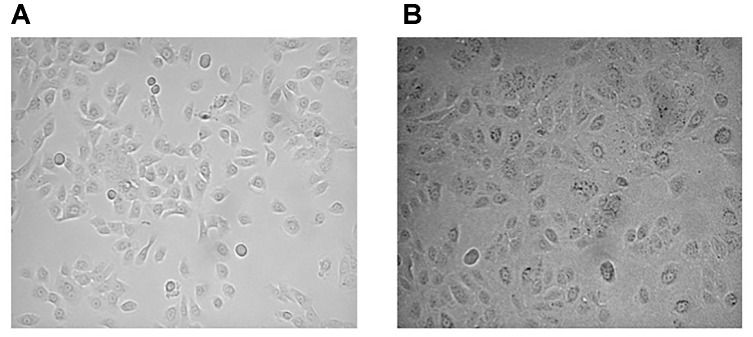
Epithelial cell culture density in the proliferative phase. Epithelial cells with a good morphology and a high proliferation rate **(A)**. Epithelial cells with an increasing number of large squamous cells and decreased cell growth **(B)**.
5. Measure transepithelial electrical resistance and potential difference with a voltohmmeter to check differentiation of polarized epithelia. We have observed that after 6 days of culture, the value of potential difference in CF epithelia is larger than non-CF.
6. The following remarks contribute to the development of polarized epithelia:
  • Detach cells at the proper growth density.
  • Seed the proper amount of cells on a permeable support.
  • Replace differentiation medium every day.

### Recording Transepithelial Ion Transport

The airway epithelium balances Na^+^ absorption and Cl^-^ secretion to carefully regulate the properties of the ASL in order to ensure efficient mucociliary clearance.

Here we present electrophysiological approaches to perform transepithelial ion transport measurements. Moreover, we highlight the applications, the strengths, and limitations of the techniques.

#### Short-Circuit Current Recordings Using the Ussing Chamber Technique

The Ussing chamber provides a system to measure the transport of ions across epithelial tissues.

Although it can be complicated to perform, it is considered the gold standard for electrophysiological experiments with the aim of studying epithelial transport.

In the most used configuration, the short-circuit current (Isc) technique, the transepithelial potential difference is clamped at zero with a voltage clamp amplifier and the resulting current is recorded.

The Ussing system consists of two hemi-chambers separated by the monolayer of epithelial cells grown on Snapwell supports, a perfusion system, an amplifier, and a data acquisition system.

Both hemichambers are fill with a Ringer’s solution, continuously bubbled with a gas mixture containing 5% CO_2_ -95% air that maintains the physiological pH. The temperature of the system is kept at 37°C. The voltage-clamp is connected to the apical and basolateral chambers with Ag/AgCl electrodes and agar bridges (two for voltage and two for current). The short-circuit current is recorded with a PowerLab 4/25 analog-to-digital converter connected to a computer.

A typical experiment is conducted as follows:

1. Fresh culture medium is replaced the day before the experiment. The medium may contain drugs or compounds. For example, epithelia from F508del patients may be treated with 1 μM lumacaftor to rescue mutant CFTR protein. As a control, cells are treated with an equal amount of control vehicle (usually DMSO at 0.1%; higher amounts of DMSO may significantly alter epithelial properties).
2. Snapwell support, carrying differentiated bronchial or nasal epithelia, is mounted in a vertical diffusion Ussing chamber.
3. Add 5 ml/hemichamber of warm Ringer’s solution and connect each side with the latex tubes that supply the gas mixture for solution bubbling.
4. Insert the current and voltage electrodes into each hemichamber and connect to the voltage-clamp amplifier (follow the instructions of the amplifier manufacturer for correct procedures).
5. Under open-circuit conditions, measure the spontaneous transepithelial electrical potential difference. Then switch the amplifier to voltage-clamp configuration and measure the resulting Isc. Calculate the transepithelial electrical resistance from the values of the transepithelial electrical potential difference and the short-circuit current with Ohm’s law. Low resistance values (below 500 Ω) may reflect a “leaky” epithelium that might increase paracellular ion flux.
6. Start recording and allow the transepithelial current to stabilize (10 min) before adding stimuli.
7. Add 10 μM amiloride to apical side to block the ENaC channel. The amplitude of the current drop caused by amiloride is dependent on the expression/function of ENaC. However, it should be kept in mind that experiments are carried out on an intact epithelium. Therefore, transepithelial ion transport is dependent on the coordinated activity of different ion channels and transporters. Changes in amiloride-sensitive current may also occur following modified expression/function of proteins that affect intracellular ion concentrations and membrane potential.
8. Stimulate CFTR channel by adding 5 μM forskolin or 10 μM CPT-cAMP (apical and basolateral sides). Both agents activate CFTR as cAMP agonists therefore they stimulate transepithelial Cl^-^ and bicarbonate transport. Anion secretion may be also promoted by activation of cAMP-dependent basolateral proteins (e.g., K^+^ channels) that generate the required electrochemical driving force.
9. In the case of epithelia from CF patients with CFTR channel gating mutations, add 20 μM forskolin or 100 μM CPT-cAMP in order to maximize CFTR activation, and subsequently add a potentiator compound on apical side (e.g., 50 μM genistein or 1 μM ivacaftor) to further stimulate channel opening.
10. Once the CFTR-dependent current is stabilized, add the CFTR blocker CFTR_inh_-172 (10 μM). The amplitude of the current drop elicited by the inhibitor reflects the extent of CFTR function. However, as discussed for ENaC, other proteins also contribute to CFTR-depedent anion secretion.
11. In the presence of amiloride and CFTR_inh_-172, the activity of other channels can be recorded. For example, stimulation with Ca^2+^ agonists (e.g., apical UTP or ATP) elicits anion secretion through Ca^2+^- dependent chloride channel (ANO1, a.k.a.TMEM16A).
12. Stop the recording and save the trace.

#### Ussing Chamber Technique Application

Using *ex vivo* airway epithelia, this technique gives a representation of the functional characteristics and processes involved in the physiological environment.

1. Measurement of physiological ion transport in non-CF epithelia.
2. Study of alterations to ion transport occurring in CF or other pathologies.
3. Evaluation of therapeutic interventions.

#### Important Remarks

1. Ussing chamber is a valuable technique but its disadvantages include technical expertise, the cost, and space needed for the equipment.
2. It is limited to polarized tissue and it does not allow analysis of a large number of samples, thus this technique has a relatively low throughput.
3. Ussing chamber is not suitable for measuring electroneutral transporters such as the Cl^-^/HCO_3_^-^ exchanger (e.g., pendrin protein).
4. Ussing chambers are made of plastic polymers, therefore some compounds used in the experiments (particularly those that work at very low concentrations such as ivacaftor) can penetrate plastic surfaces and be released in subsequent experiments thus altering the results. To overcome this contamination problem, we recommend that the chambers be washed at the end of each experiment in a sodium phosphate tribasic solution (15 g diluted in 1 l of deionized water) for 4 h and then in hydrochloric acid 0.1% solution for 15 min. After that, rinse in deionized water.
  Agar salt bridges represent another source of contamination. We suggest that the bridges be replaced at the end of each experiment.

#### Electrical Epithelial Properties Measured With the Trans-Epithelial Electrical Resistance (TEER) – Potential Difference (PD) Technique

TEER/PD is a rapid and simple method to quantify ion transport by measuring the electrical resistance and potential difference across the epithelium. TEER/PD is dependent on the expression/activity of channels situated on both apical and basolateral membranes as well as on the properties of tight junctions that control paracellular ion transport.

TEER/PD measurements are performed using the EVOM2 system and placing Ag/AgCl “chopstick” electrodes on both sides of an epithelial monolayer cultured on HTS Transwell permeable supports.

The method is simply based on the separate measurement of TEER and PD values for each epithelium, under resting conditions and following the application of agonists and inhibitors. The equivalent short-circuit current is then calculated from TEER and PD.

Considering the simplicity of the measurement and the data reproducibility compared with the Ussing chamber method, TEER/PD measurements are a good benchmark tool for high-throughput drug discovery screening in CF epithelia.

1. Culture bronchial or nasal epithelial monolayers in HTS Transwell inserts as previously described.
2. Replace the medium the day before the experiment. If needed, treatments can be included (e.g., lumacaftor or other correctors for CF epithelia).
3. Wash the chopstick electrodes with deionized water and equilibrate the electrodes in a Coon’s modified solution.
4. Incubate both sides of the epithelia with Coon’s modified solution for 1 h at 37°C.
5. Measure TEER and PD under basal conditions.
6. Add 10 μM amiloride solution on the apical side. Blocking ENaC should result in a higher TEER and lower PD due to inhibition of the sodium-dependent current.
7. Wait 10 min at 37°C before recording measurements.
8. Replace the apical and basolateral solution with new solution containing the CFTR activating cocktail (5–20 μM forskolin and, if needed, 50 μM genistein). The apical side should also contain amiloride. Wait 10 min before taking the measurement. It should be noted that genistein is preferred as a low potency CFTR potentiator instead of the high potency ivacaftor. Ivacaftor may stick to electrodes and affect subsequent experiments.
9. The final measurement is performed by adding 30 μM PPQ-102, a CFTR inhibitor, on the apical side. This is done by replacing the apical solution with a new one containing PPQ-102 together with amiloride and a CFTR-activating cocktail at the indicated concentrations. The change in equivalent current elicited by the inhibitor reflects the extent of CFTR function.
10. Wash electrodes with deionized water and 70% ethanol solution.

#### Important Remarks

1. In the TEER method, CFTR-inh172 does not significantly reduce conductance. We have tested different inhibitors and we have observed that PPQ-102 is the best one for completely blocking CFTR activity (Tradtrantip et al., 2009).

### Fluid Absorption Rate Measurements

In CF airways defective ion transport causes a simultaneous reduction of fluid, chloride, and bicarbonate secretion. The altered properties of mucus may be studied in order to understand the pathological mechanisms and to evaluate treatments for recovering hydration of the airways.

Epithelia with serum-free medium reproduces ASL periciliary characteristics with mucus and fluid secretion.

In this section, we present a protocol that allows the measurement of fluid absorption rate.

1. Epithelia begins to secrete mucus and fluids on the apical side after nearly 15 days of culture on Snapwell supports. A fully differentiated epithelia is obtained after 4 weeks of culture.
2. After 15 days of culture, wash the apical surface once a week with D-PBS to remove secreted mucus.
3. Use some of the epithelia of the same cell preparation for short-circuit current recordings to check appropriate ion transport.
4. Apply 130 μl D-PBS on the epithelium surface. We suggest that the apical fluid to recovered between 3 and 48 h after D-PBS incubation.
5. Weigh an empty 1.5 ml collection tube (tare).
6. Carefully collect the fluid remaining after incubation with a pipette and place it in the collection tube (Figure 3).

**FIGURE 3:**
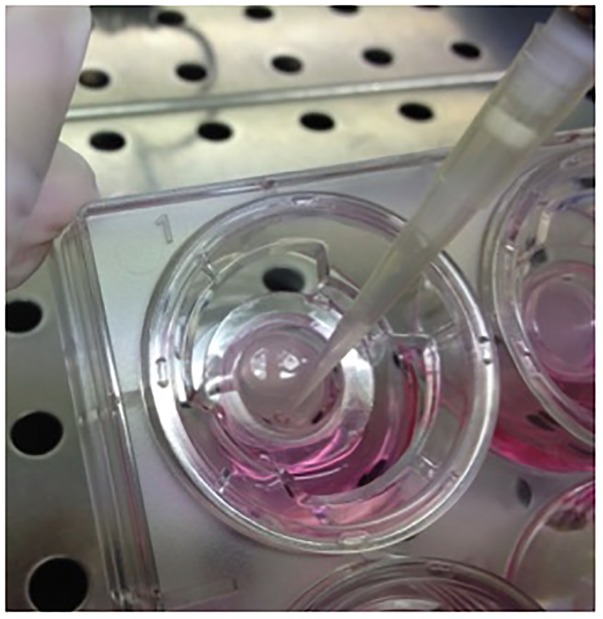
Collection of fluid and mucus secreted from differentiated epithelia. Periciliar fluid collection from surface epithelia after D-PBS incubation.
7. Weigh the tube again and estimate the final apical volume collected:
  Vf = (Wf-Wi) × 000/N. Where Wf = tare and collected fluid weight; Wi = tare weight; N = number of pool samples.
8. Calculate fluid transport rate: Jw = (Vi-Vf)/A × t. Where Vi = initial apical volume (130 μl); Vf = final apical volume; A = area of the epithelium (for Snapwell insert: 1.13 cm^2^); t = time interval.

#### Important Remarks

1. During the first days of ALI culture, it is normal to observe some liquid (from the basolateral medium) on the epithelium surface due to the fact that the tight-junctions are not completely formed. This condition will persist in a leaky epithelium.
2. CF secreted mucus is more viscous and difficult to pipette compared to non-CF mucus.

## Anticipated Results

To overcome the scarcity of biological material available for primary culture, our group has developed a protocol to extract primary airway epithelial cells from lung resections or nasal brushing and subsequently culture and amplify these cells, maintaining most of the characteristics of the airway epithelium *in vivo*.

The procedure starts by using a proliferative medium that promotes cell growth at a low density and a high proliferation rate in order to obtain a large number of polarized bronchial and nasal epithelia useful for the study of CF physiopathology.

This is followed by a differentiation phase. We have presented two protocols that both require seeding cells on permeable supports and the “ALI” condition, but they differ depending on the final aim of the designed experiments.

The serum-free medium protocol provides a fully differentiated epithelial monolayer that is useful for evaluating morphological and periciliary fluid properties in primary airway epithelia. However, a critical point of this protocol is the prolonged culture time and the cost of medium supplements.

Instead, we suggest that the serum-complemented medium be applied to the protocol for functional electrophysiologic assays, because it still provides a differentiated monolayer with transepithelial ion transport properties similar to *in vivo* conditions, but in a shorter time and with lower costs compared to the serum-free medium protocol. Therefore, this medium is optimal for functional electrophysiologic assays.

To compare the two protocols for evaluating epithelial morphological properties, we have used immunofluorescence combined with confocal microscopy. This technique provides high-resolution imaging of protein localization, expression patterns, and different cell types that characterize the airway of mature epithelium.

Cultured bronchial epithelia are analyzed by immunofluorescence for acetylated tubulin and MUC5AC to identify ciliated and goblet cells, respectively. To distinguish the apical from the basolateral membrane we have used an antibody against the ZO-1 tight junction marker.

**FIGURE 4:**
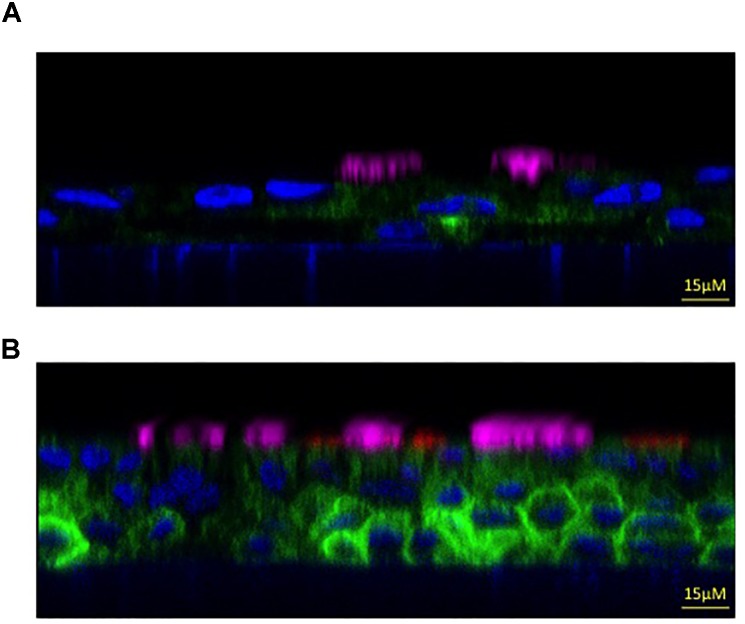
Morphologic properties showing a mature airway epithelium. Representative *xz* immunofluorescence images taken with a confocal microscope (Leica SP8 Laser Scanning Confocal Microscope) to show non-CF bronchial epithelia differentiated with serum-supplemented medium **(A)** or with serum-free medium **(B)**. Cilia are labeled in magenta (anti-tubulin antibody), the goblet cells in red (anti-MUC5AC antibody), the apical membrane in green (anti-TMEM16A antibody) and the nucleus in blue (Hoechst dye). Images kindly provided by Dr. Paolo Scudieri (TIGEM, Pozzuoli, Italy).

The derived staining shows that epithelium differentiated with serum-supplemented medium is pseudo-stratified with a relatively small number of ciliated and goblet cells (Figure 4A).

On the contrary, when using the serum-free medium protocol we obtained a higher pseudo-stratified epithelium characterized by a higher number of ciliated and goblet cells (Figure 4B). This result demonstrates that the latter culture condition provides most of the morphological characteristics of the airway epithelium *in vivo*, thus we suggest that it be used to analyse the expression and localization of selected proteins and airway cells.

In the CF lung, the decrease in CFTR-mediated chloride and fluid secretion are believed to cause the dehydration of the airway surface by altering the properties of periciliary mucus. To gather significant information, we have applied the serum-free medium protocol, that reproduces ASL periciliary characteristics with mucus and fluid secretion, to analyse the rate of fluid absorption of non-CF and CF airway mucus recovered from cultured HBEC.

Time course experiments confirmed that the different activities of CFTR in non-CF and F508del-CFTR epithelia resulted in significant differences in the net absorption rate after 24 and 48 h; reflecting the different ASL characteristics.

**FIGURE 5:**
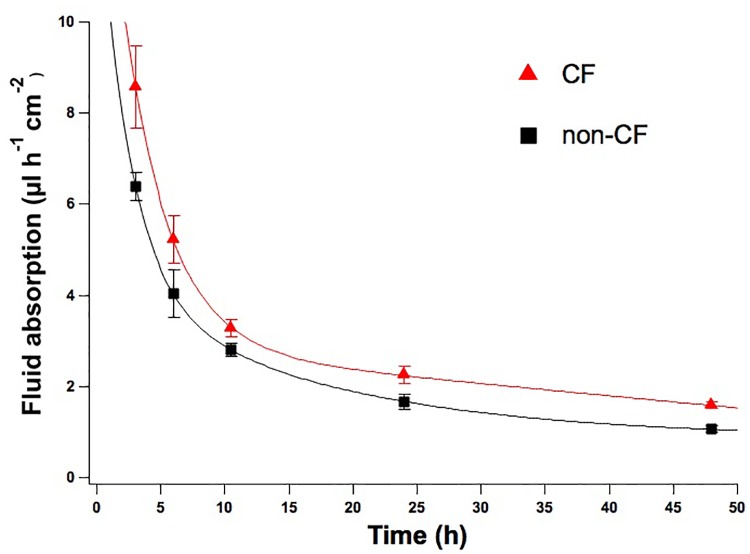
Time course rate of fluid absorption of non-CF and CF epithelia. Net fluid absorption rate measured on non-CF (black squares) and CF (red triangles) epithelia up until 48 h of incubation with D-PBS. Mean values ± SEM are shown, at the timepoints of 24 and 48 h the differences between CF and non-CF epithelia are significant (*p* < 0.05).

While non-CF epithelia absorbed after 24 and 48 h 1.67 ± 0.15 μl/h/cm^2^ (*n* = 5) and 1.06 ± 0.06 μl/h/cm^2^ (*n* = 7), respectively, in F508del-CFTR epithelia net absorpion rate was 2.25 ± 0.19 μl/h/cm^2^ (*n* = 5) and 1.58 ± 0.07 μl/h/cm^2^ (*n* = 7) (Figure 5).

Moreover, during recovery we observed that the CF fluid is more viscous and difficult to pipette compared to non-CF.

The development and validation of CF-HBEC aims to support drug discovery efforts to correct basic CF defects. For this purpose, electrophysiological approaches allow transepithelial ion transport to be measured in order to assess the efficacy of pre-clinical CFTR modulators in primary human airway cultures.

We have used the Ussing chamber technique to measure short-circuit current as an indication of net ion transport across bronchial and nasal epithelia monolayers maintained in serum-supplemented medium.

**FIGURE 6:**
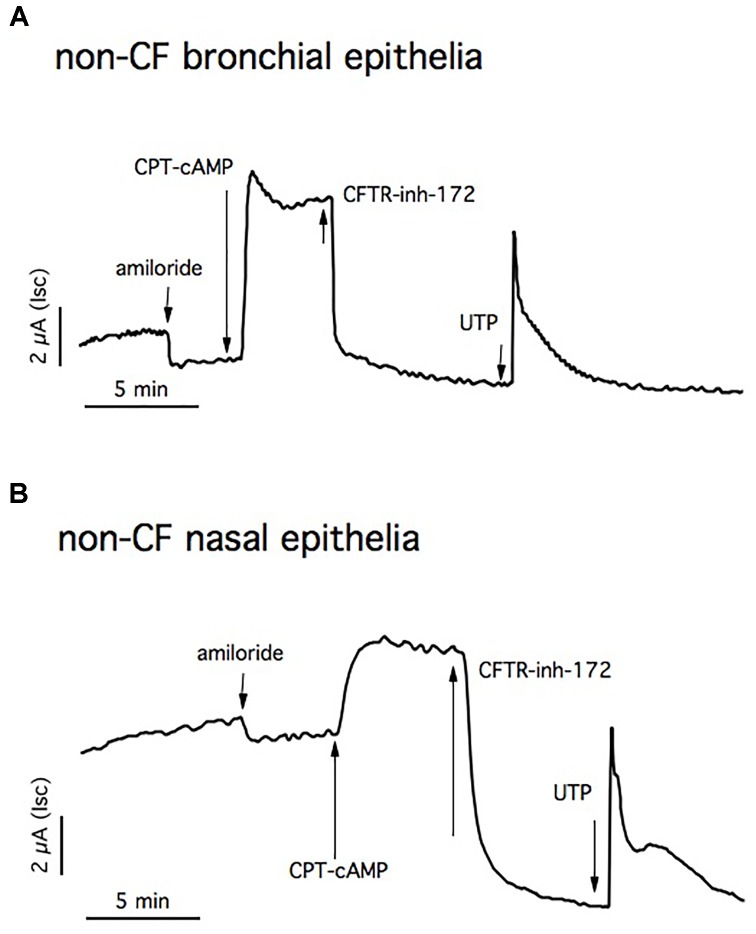
Short circuit currents (Isc) measurements in non-CF epithelia. Representative traces from Ussing chamber recordings of non-CF bronchial **(A)** and nasal epithelia **(B)**. The ENaC channel is blocked with apical amiloride and CFTR activation and inhibition is obtained by adding CPT-cAMP and CFTR_inh_-172, respectively. The Ca^2+^- dependent chloride channel is stimulated with UTP.

Figure 6A shows a representative trace experiment of short-circuit currents of differentiated non-CF bronchial epithelia. The basal current was inhibited by amiloride, indicating the presence of epithelial Na^+^ channel (3.1 ± 0.6 μA/cm^2^; *n* = 25). To assess the amount of CFTR-dependent Isc, cells are blocked with CFTRinh-172 after maximum CFTR activation using CPT-cAMP. The resulting chloride current is strongly sensitive to CFTRinh-172 (10.08 ± 0.04 μA/cm^2^; *n* = 31). Application of UTP generates a very fast calcium-activated chloride current (3.8 ± 1 μA/cm^2^; *n* = 29).

Primary human nasal monolayers recapitulate the transepithelial ion transport properties of bronchial cells, confirming the fact that this cell model can predict clinical treatment efficacy in patients (Figure 6B).

**FIGURE 7:**
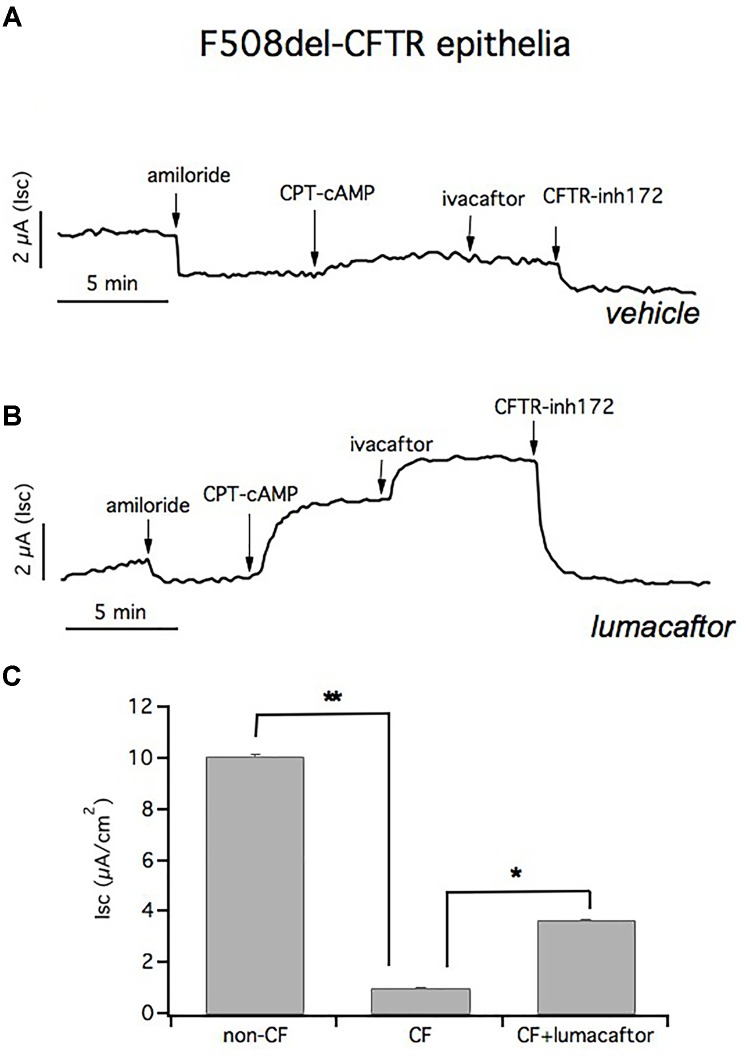
Short circuit currents (Isc) in CF and in lumacaftor-treated CF epithelia. Representative traces from short circuit current (Isc) recordings of homozygous F508del-CFTR epithelia treated with vehicle **(A)** and lumacaftor **(B)**. The ENaC channel is blocked with apical amiloride and CFTR activation and inhibition is obtained by adding CPT-cAMP plus ivacaftor and CFTR_inh_-172, respectively. **(C)** Graph bars summarizing CFTR-mediated currents from Ussing chamber recordings of HBEC derived from 4 non-CF donors (*n* = 22) and from 4 homozygous F508del-CFTR patients (*n* = 41). Mean values ± SEM are shown, ^∗^ and ^∗∗^ indicate *p* < 0.05 or *p* < 0.001, respectively.

To further support the conclusion that our culture protocol allows F508del-CFTR-HBEC to reflect the ion transport defects in airway epithelia *in vivo*, we used cells from patients that were homozygous for this mutation; which causes a severe defect in CFTR function. As expected the response to CPT-cAMP and ivacaftor is markedly reduced in negative control cells (1.04 ± 0.023 μA/cm^2^; *n* = 37) (Figure 7A). However, we have noted that lumacaftor treatment for 24 h induced a significant increase in chloride transport, as indicated by the current blocked by CFTRinh-172 (3.67 ± 0.07 μA/cm^2^; *n* = 41) (Figure 7B).

Figure 7C summarizes the CFTR-mediated currents from Ussing chamber recordings of HBEC derived from 4 non-CF donors and 4 homozygous F508del-CFTR patients. Transepithelial current measurements showed that there was a significant difference in the CFTR-mediated Isc in cultured F508del-HBE cells compared to non-CF HBE cells, and incubation of CF epithelium with lumacaftor led to a significant improvement in CFTR-dependent chloride secretion.

TEER is a quantitative technique that is useful for predicting the ideal concentration of a drug to be tested, without damaging the epithelium, or to quantify ion transport by measuring the electrical resistance and potential difference across the barrier tissue.

We have performed TEER measurements to evaluate the ability of compounds to correct F508del-CFTR. Bronchial epithelia from patients that were homozygous for the F508del mutation were treated for 24 h with lumacaftor, or vehicle (DMSO), and then assayed. Transepithelial electrical resistance was measured before and after acute stimulation with forskolin and genistein to fully activate CFTR. Subsequently, transepithelial resistance was measured after the addition of the selective CFTR inhibitor, PPQ-102, used to completely block CFTR activity. The delta between the values of electrical resistance before and after CFTR inhibition were then converted into the reciprocal conductance.

**FIGURE 8:**
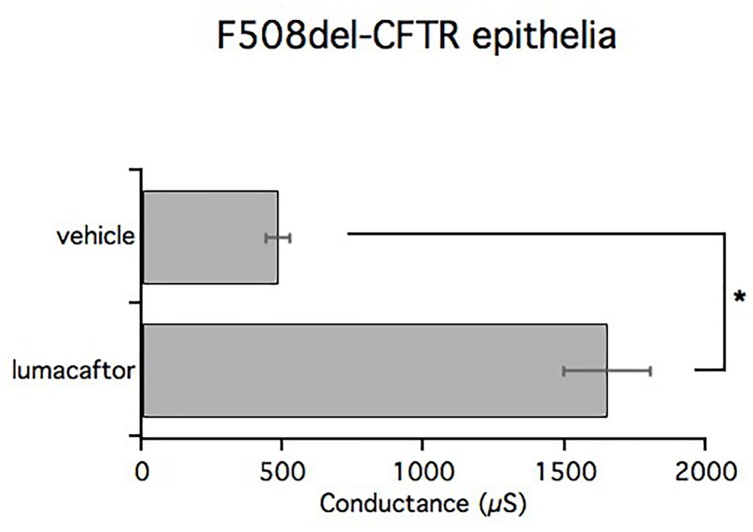
TEER measurements in CF and lumacaftor-treated CF. Graph bars indicate TEER measurements performed on homozygous F508del-CFTR epithelia derived from 4 homozygous F508del-CFTR patients (*n* = 12) treated with vehicle or lumacaftor. The bar graphs show the electrical resistance values measured before and after CFTR inhibition, converted into their reciprocal conductance. Mean values ± SEM are shown, ^∗^ indicate *p* < 0.05.

In accordance with short-circuit current measurements, treatment with lumacaftor significantly increased transepithelial conductance (1650 ± 153.3 μS/cm^2^) compared with the negative control (482 ± 42.2 μS/cm^2^). The bar graph shows mean values obtained from four different omozygous F508del-CFTR patients (Figure 8).

In conclusion, we have provided evidence that our procedures provide highly differentiated airway epithelial monolayers with morphologic and functional characteristics similar to airway epithelia *in vivo*. These cell models are very useful for improving our knowledge about physiopathology mechanisms involved in CF and to support therapeutics strategies.

## Ethics Statement

The procedure of the bronchus dissection and the informed consent from patients was approved by the Ethical Committee of the Gaslini Institute under the supervision of the Italian Ministry of Health.

## Author Contributions

AG and LD have performed the procedures and contributed equally to the work. EC has written the manuscript with the help of AG and LD.

## Conflict of Interest Statement

The authors declare that the research was conducted in the absence of any commercial or financial relationships that could be construed as a potential conflict of interest.
