# Supplementary material for: In vitro Methods for the Development and Analysis of Human Primary Airway Epithelia
Source: Front Pharmacol. 2018 Oct 26;9:1176. doi: 10.3389/fphar.2018.01176 (PMC6212516; doi:10.3389/fphar.2018.01176)
Supplement: Supplementary file 1 [file Table_1.docx]

Supplementary Material

### *IN VITRO* methods For the DEVELOPMENT and ANALYSIS OF HUMAN PRIMARY AIRWAY EPITHELIA

Ambra Gianotti, Livia Delpiano and Emanuela Caci *****.

U.O.C. Genetica Medica, IRCSS Istituto Giannina Gaslini, Genoa, Italy.

***Correspondence:**Corresponding Author:

[emanuela.caci@unige.it](mailto:emanuela.caci@unige.it)

**LIST OF SUPPLEMENTS AND PREPARING FOR BRONCHIAL AND NASAL EPITHELIAL CELLS CULTURE**

- BSA stock: dissolve 1 mg/ml bovine serum albumin (Sigma #A8806) in HBSS. Sterilize with a filter, store at –20 °C in aliquots.
- Calcium stock: dissolve 116 mM calcium chloride dehydrated (Sigma #C3881) in deionized water. Sterilize with a filter, store at +4 °C for months.
- E stock: dilute 0.015 ml ethanolamine (Sigma #E0135) in 2.5 ml BSA stock and 22.5 ml HBSS. Store at -80 °C in aliquots.
- Epidermal growth factor stock (EGF): dissolve 5 μg/ml EGF-human recombinant (Roche, 11376454001, Basilea, Switzerland) in 4 ml BSA stock and 36 ml HBSS. Store at –80 °C, do not refreeze after thawing.
- Epinephrine stock: dissolve 1 mg/ml epinephrine (Sigma #E4250) in 10 mM hydrochloric acid. Store at -80 °C in aliquots.
- Hydrocortisone stock (HC): dissolve 3.6 mg/ml hydrocortisone (Sigma #H0888) in 10 mM ethanol absolute. Store at -80 °C in aliquots.
- Insulin stock: dissolve 5 mg/ml bovine insulin (Sigma #I1882) in 10 mM in hydrochloric acid. Store at –80 °C in aliquots.
- Manganese stock: dissolve 0.0394 mg/ml manganese (II) chloride tetrahydrate (Sigma #M3634) in deionized water. Sterilize with a filter and store at +4 °C for months.
- Molybdenum stock: dissolve 0.124 mg/ml ammonium molybdate (Sigma #A1343) in deionized water. Sterilize with a filter and store at +4 °C for months.
- Nickel stock: dissolve 0.052 mg/ml nickel (II) sulfate hexahydrate (Sigma #227676) in deionized water. Sterilize with a filter and store at +4 °C for months.
- P stock: dissolve 28.5 mg phospoethanolamine (Sigma #P0503) in 2.5 ml BSA stock and 22.5 ml HBSS. Store at -80 °C in aliquots.
- P/E stock: add 0.125 ml P stock and 0.125 ml E stock to 12.25 ml HBSS. Store at +4 °C for no longer than one month.
- Retinoic Acid stock (RA): dissolve 1 mg/ml retinoic acid (Sigma #T6397) in DMSO. Store at -80 °C in aliquots.
- RA/T_3_ stock: add 5µl T_3_ stock and RA stock to 0.5 ml DMSO. Prepare before using, do not store.
- Selenium stock: dissolve 0.26 mg/ml sodium selenite (Sigma #5261) in deionized water. Sterilize with a filter and store at +4 °C for months.
- Silicone stock: dissolve 0.052 mg/ml sodium metasilicate nonahydrate (Sigma #S4392) in deionized water. Sterilize with a filter and store at +4 °C for months.
- Stock 4: dissolve 42 mg ferrous sulfate heptahydrate (Sigma #F7002), 12.2 grams magnesium chloride hexahydrate (Sigma #M2670), 333 mg calcium chloride dehydrate (Sigma #C3881) and 0.5 ml hydrochlorid acid 37% in 1 liter deionized water. Sterilize with a filter and store at +4 °C for months.
- Stock 11: dissolve 0.863 mg/ml zinc sulfate heptahydrate (Sigma #Z0251) in deionized water. Sterilize with a filter and store at +4 °C for months.
- Tin stock: dissolve 0.044 mg/ml stannous chloride dihydrate (Sigma #431508) in deionized water. Sterilize with a filter and store at +4 °C for months.
- Trace elements solution: add 50 µl of the following stock solutions: manganese stock, molybdenum stock, nickel stock, selenium stock, silicone stock, tin stock and vanadium stock to 50 ml deionized water. Sterilize with a filter and store at +4 °C for months.
- Transferrin stock: dissolve 100 mg human transferrin (Sigma #T8158) in 1 ml BSA stock and 9 ml HBSS. Store at -80 °C in aliquots.
- Triiodothyronine stock (T_3_): dissolve 0.65 mg/ml triiodothyronine (Sigma #T6397) in n-propanol 50%. Store at -80 °C in aliquots.
- Vanadium stock: dissolve 0.118 mg/ml ammonium metavanadate (Sigma #204846) in deionized water. Sterilize with a filter and store at +4 °C for months.
